# Stereotactic Radiosurgery for the Treatment of Recurrent High-grade Gliomas: Long-term Follow-up

**DOI:** 10.7759/cureus.6527

**Published:** 2019-12-31

**Authors:** Kita Sallabanda, Loreto Yañez, Morena Sallabanda, Marcos Santos, Felipe A Calvo, Hugo Marsiglia

**Affiliations:** 1 Radiosurgery/ Neurosurgery, Hospital Clinico Universitario San Carlos, Madrid, ESP; 2 Radiotherapy, Fundación Arturo López Pérez, Rancagua, CHL; 3 Radiation Oncology, Hospital Universitario Puerta de Hierro, Madrid, ESP; 4 Neurosurgery, Instituto Madrileño De Oncología, Madrid, ESP; 5 Radiation Oncology, University of Navarra, Madrid, ESP; 6 Radiation Oncology, Fundación Arturo López Pérez (Santiago de Chile), Santiago de Chile, CHL

**Keywords:** radiosurgery, cyberknife®, recurrent gliomas, recurrent gliomas, recurrent gbm, srs, ck-cyberknife, glioblastoma multiforme (gbm)

## Abstract

High-grade gliomas (HGG) are the most frequent primary central nervous system tumors; treatment of HCGs includes surgery and post-operative conformal radiotherapy associated with temozolomide (TMZ or procarbazine/lomustine/vincristine [PCV], specifically in patients with anaplastic oligodendrogliomas or anaplastic oligoastrocytomas). However, recurrence is common. Re-irradiation has been utilized in this setting for years and remains a feasible option, although there is always a concern regarding toxicity. Modern high-precision conformal techniques, including stereotactic radiosurgery (SRS), could improve the therapeutic ratio by delivering high biologically equivalent doses while reducing high-dose radiotherapy (RT) to normal brain tissue. In this paper, we present the results obtained after prolonged follow-up in patients who underwent SRS as a treatment for recurrent high-grade gliomas at San Francisco Hospital in Madrid, Spain

## Introduction

High-grade gliomas (HGG) are the most frequent malignant primary central nervous system tumors in adults, accounting for more than 20% of all primary brain neoplasms [1]. Surgery and post-operative conformal radiotherapy (RT) associated with temozolomide (TMZ or procarbazine/lomustine/vincristine [PCV], specifically in patients with anaplastic oligodendrogliomas or anaplastic oligoastrocytomas) have become the cornerstone of therapeutic approaches. However, despite recent advances in treatment options, local recurrence continues to be a common event.

The first challenge is the differential diagnoses between glioma recurrence and radiation necrosis, which is crucial since the two entities have different treatment approaches and prognosis. Computed tomography (CT) and magnetic resonance imaging (MRI) suffer from significant limitations in this setting [2]. Depending on the clinical follow-up to make the distinction between radionecrosis and recurrence is a common practice; however, this raises concerns, because in more than 30% of patients, radiation necrosis presents itself as swelling or as a mass which results in clinical deterioration, requiring resection. Once recurrence is confirmed or suspected, surgical resection is often the first option to alleviate symptoms, followed by salvage systemic treatment or/and reirradiation [3]. Total tumor surgical resection can be difficult and almost impossible to achieve due to the extensive parenchymal infiltration, the anatomical location, and most importantly, the patient's own decision-making process regarding the willingness to undertake risks of resection in the context of short survival expectation. Moreover, this aggressive strategy usually achieves modest and transient results and should be reserved to a very specific subgroup of patients [3-4]. Concerning systemic treatment, available regimens are often limited by patients’ poor condition, tumor resistance, poor blood-brain barrier penetration, and significantly compromised overall patient functional status. Re-challenging with TMZ or switching to a non-conventional TMZ regimen (for example, metronomic TMZ) has become a common strategy [5]. More recently, several targeted therapies have been introduced in clinical practice and investigated in clinical trials with uncertain results concerning safety, with no clear evidence of benefit; antiangiogenic therapy with bevacizumab improves progression-free survival, without a significant benefit in overall survival improvement [2,6].

Re-irradiation has been utilized in this setting for years and remains a feasible option, although there is always the concern of a relatively high risk of toxicity [7-12]. Modern high-precision conformal techniques, including stereotactic radiosurgery (SRS), have the power to improve the therapeutic ratio by delivering high biologically equivalent doses while reducing high-dose RT to normal brain tissues [7]. In this paper, we present the results obtained after prolonged follow-up in patients who underwent SRS as a treatment for recurrent high-grade gliomas at the Neurosurgery department at San Francisco Hospital in Madrid, Spain.

## Materials and methods

A total of 50 patients with recurrent HGGs (Grade III and IV) were retrospectively reviewed after being treated with SRS at the Department of Neurosurgery in the San Francisco Hospital, Madrid, Spain from January 1992 to December 2014. There were 36 men (72%) and 14 women (28%). The entire study population had received surgery and adjuvant radiotherapy as the first course of treatment. Surgery was performed on one patient, and in 23 patients (46%), only a biopsy was performed. Glioblastoma multiforme was the diagnosis of 48% of the patients, while 40% had anaplastic astrocytomas and 12% had anaplastic oligodendrogliomas. Most of the patients received systemic treatment (72%), TMZ being the most frequently used drug (58%). At recurrence, patients presented behavior disturbances (48%), motor deficits (46%), sensitivity deficits (24%), speech disorders (22%), and seizures (16%; Table 1).

**Table 1:** Patient characteristics

Variable		n	%
Sex	Men	36	72%
	Women	14	28%
Surgery	R0	1	2%
	Partial resection	26	52%
	Biopsy	23	46%
Histology	Glioblastoma	24	48%
	Anaplastic astrocytoma	20	40%
	Oligodendroglioma	6	12%
Chemotherapy	Temozolomide	29	58%
	Others	6	12%
Behavior disturbances	Yes	24	48%
	No	26	52%
Motor Deficit	Yes	23	46%
	No	27	54%
Sensitivity Deficit	Yes	12	24%
	No	38	76%
Speech disorders	Yes	11	22%
	No	39	78%
Seizures	Yes	8	16%
	No	42	84%

The mean age of the whole group was 51.5 years (21-81 years). With regard to radiation treatment, the mean delivered dose was 59.5 Gy (range: 40-76 Gy), and three patients received intraoperative radiation boost (IORT) with doses varying from 6 to 12.5 Gy, according to the institutional protocol at that time [8]. The mean interval between first treatment and recurrence was 23.2 months (range: 1 to 196 months), with this interval being shorter in glioblastoma patients (mean: 13.5 months, range: 1-60 months) and larger in oligodendroglioma patients (mean: 54.7 months, range: 9-196 months). The mean follow-up was 55.5 months (range: 11-357 months). Response assessment in neuro-oncology criteria (RANO) criteria has been used to evaluate the imagenologic recurrence.

Since it is a broad retrospective series, the state of methylation (methylguanine DNA methyltransferase; MGMT) or isocitrate dehydrogenase (IDH) in the treatment regimen is not taken into account and has not been collected in several of the cases.

Procedures

In all cases, SRS was carried out using a linear accelerator with a high-precision positioning system and mechanical fixation of the tertiary collimator (SRS 200; University of Florida, Gainesville, FL), with 6-MV photons. To locate the lesion, MRI was performed after which the stereotactic frame was placed under local anesthesia for the planning CT phase. An image fusion program was used to fuse imaging datasets and delimit the target volume. During the study period, different treatment planning systems were utilized. Three-dimensional treatment planning was made in all cases, using different planning units (Philips SRS 200 [Philips, Madison, WI], Brain Lab [Brain-Lab, Feldkirchen, Germany], Plato-Nucletron [Nucletron, Veenendaal, Netherlands], and ERGO-3D Line [3DLine Medical Systems, Milan, Italy]) during the period of the study.

Dose planning was performed to cover target only the MRI enhancing tumor as conformational as possible. Dose prescription took into consideration the following variables: previous doses delivered during the first course of treatment, the interval between treatment and recurrence and proximity to critical structures, such as the optic nerve, optic chiasma, or brainstem, and the clinical situation of the patient. A median dose of 14 Gy (range: 8-20 Gy) was prescribed to the 90% (range: 50% to 100%) mean isodose surface. After SRS, all patients were subjected to receive prophylactic treatment with dexamethasone and were observed in the hospital for 24 hours to prevent any early complications. Tumor size before and after SRS was assessed by measuring the contrast-enhanced margins in the three standard MRI dimensions. A follow-up MRI, with and without gadolinium enhancement, was carried out after six and 12 months and yearly thereafter. MRI protocols have evolved over the period of the study, along with the evolutionary implementation of MRI technology, as did radiosurgically induced change interpretation.

Statistical analysis

The median survival was the survival defined as the time from the procedure (SRS) until the onset of clinical progression or death. All eligible cases were included in the survival analysis. The estimation of the cumulative proportion surviving was based on Kaplan-Meier procedures. To analyze factors correlating with survival, the following parameters were assessed: age (50 years vs. >50 years), sex, and histology (oligodendroglioma vs. anaplastic astrocytoma vs. glioblastoma). SPSS version 12.0 (SPSS Inc., Chicago, IL) was used to analyze the results.

## Results

The median overall survival (OS) of the whole group was 11 months (95% CI: 7.3 to 14.6 months). Around half of the patients died by the end of the first year after radiosurgery (50.9%), and 17% were alive by the end of the third year. Following SRS, 29 of our patients received adjuvant systemic chemotherapy, as per the medical oncologist’s preference order following SRS.

There were no differences in median survival when comparing male to female patients by gender. Patients under the age of 50 years showed no differences in OS when compared to their older counterparts, although a tendency to an improved OS was noted in the younger population. 

When considering the histological type as expected, we observed significant better median survival in anaplastic oligodendrogliomas (median survival: 91 months), anaplastic astrocytomas (median survival: 12 months; 95% CI: 7.9 to 16 months), and glioblastomas (median survival: 8 months; 95% CI: 4.1 to 11.8 months).

During the follow-up, six patients did not present toxicity following SRS, while 44 patients (88%) presented toxicities: grade I (Radiation Therapy Oncology Group; RTOG): 21 patients (asymptomatic edema in neuroimages that didn`t require treatment), grade II: 16 patients (asymptomatic edema that required short-course corticosteroid use <1 month), and grade III: seven patients (symptomatic brain edema that required prolonged use of corticosteroids).

## Discussion

The standards of care for patients with recurrent glioblastoma are not well defined, and clinical decision-making is often based on previous treatments, age, Karnofsky performance score (KPS), and patterns of relapse [3-4].

Re-irradiation, for recurrent HGG, remains a controversial treatment option [13-15].

Likewise, surgery has limited applicability, and finally, systemic therapy, if utilized alone, does not provide sufficient local control. RT has evolved to deliver higher tumor doses and reduce high-dose treatment to normal tissue around the tumor. Median survival, following recurrence, has been described to be around 25 to 30 weeks in patients with glioblastoma and between 39 to 47 weeks in patients with grade III tumors [9]. In this study, a median progression-free survival (PFS) of almost 40 months and a median OS of 41.1 months was observed, with half of the patients being alive at the end of the first year. These positive results may be explained, in part, because oligodendrogliomas are tumors that usually present a better prognosis in comparison to anaplastic astrocytomas or glioblastomas.

On the other hand, the dose/rate effect, as well as the high single dose, improves the effectiveness of radiotherapy. Based on our research and previous articles, it is clear that dose intensification is reflected clinically in an increase in local control. In our series, this is prolonged up to 26 months in patients with glioblastomas.

Regarding the high recurrence rate of HGGs, surgery remains the first option, but not all patients can be treated surgically and in many cases, we have deep tumors. Reirradiation is a good option in this group of patients, and radiosurgery has an advantage, in this sense, because it has the power to increase the dose of radiation with high precision to a determined volume in a single treatment session (or hypofractionation) when compared to conventional radiotherapy [15]. The dose gradient outside the white volume allows the preservation of surrounding critical organs. Tsao performed a systematic review in 2005, where there was insufficient evidence to make recommendations on the use of radiosurgery in the treatment of recurrent disease [10]. However, to date, there is increasing evidence to clarify this question. In our study, the median survival of 26 months in patients with glioblastoma was obtained, which is superior compared to similar series of reirradiation with IMRT, even with the use of biological agents where survival was observed 9 months after the second treatment [3-6]. It is also superior compared to the series of the patients treated with chemotherapy alone, where the reported survival rate was between 7 and 11 months and compared to series where only radiosurgery was used, with reported results between 10.9 to 32 months of OS [13-20]. The survival rates with radiosurgery observed in our series were 50.96% at one year, 17% at two years, and 6.22% at five years, which is better than a similar study using rescued radiosurgery in single or multiple sessions, in high gliomas, using a robotic accelerator [19].

Our patient complications are similar to those reported in the literature [21-22]. In our series, only one patient needed surgical treatment. Variables such as KPS, initial resection, and non-multifocality may help in the selection of patients who would benefit from local treatments such as radiosurgery, but independently, all histological varieties of high-grade gliomas benefit from this treatment strategy [23-24].

One of the main factors in determining the dose of irradiation is the ability of brain tissue to recover from sublethal damage, which is directly related to the effective biological dose of the first radiotherapy treatment and, as well, to the interval between the first treatment and radiosurgery. One study suggests that the use of extended fields, due to the infiltrative nature of the tumor, results in increased local control in small tumors. Nonetheless, it shows no difference in Simpsons grade (SG) when compared with standard radiosurgery; hence, we strongly believe that it is not necessary and SRS can protect better the normal tissues around [25].

Our study has some limitations. One of them is that it is a retrospective series. However, several similar studies have shown a benefit in the group of patients who are candidates for radiosurgery [12,26-30]. Even with this limitation, our results, in general, had better outcomes when compared to reports on previous studies, as toxicities were scarce and delaying survival, after relapse.

## Conclusions

In our study, patients with recurrent high-grade gliomas benefit from re-irradiation, with half of the patients still surviving at the end of the first year. These positive results may be explained, in part, by the presence of grade 3 oligodendrogliomas in our series. These results are comparable with other centers' experience and they support the efficacy and safety of re-irradiation in this setting. Results are better in our series if radiosurgery is used in grade 3 gliomas compared to grade 4 gliomas.

In conclusion, radiosurgery should be taken into account for the rescue of patients with recurrent high-grade gliomas. However, further studies are needed to define possible subgroups with greater benefits.
